# Causal association of metabolic syndrome with chronic kidney disease progression: A Mendelian randomization study

**DOI:** 10.1002/pdi3.93

**Published:** 2024-07-06

**Authors:** Qitong Guo, Meiling Chen, Yihang Yu, Ping Li, Xu Huang, Lianju Shen, Chunlan Long, Xing Liu, Tao Lin, Dawei He, Guanghui Wei, Deying Zhang

**Affiliations:** ^1^ Department of Urology Children's Hospital of Chongqing Medical University Chongqing China; ^2^ National Clinical Research Center for Child Health and Disorders Ministry of Education Key Laboratory of Child Development and Disorders China International Science and Technology Cooperation Base of Child Development and Critical Disorders Chongqing Key Laboratory of Pediatrics Chongqing Key Laboratory of Structural Birth Defect and Reconstruction Chongqing China

**Keywords:** chronic kidney disease, Mendelian randomization, metabolic syndrome

## Abstract

Research from the past has indicated a link between the risk of chronic kidney disease (CKD) and metabolic syndrome (MetS). It is yet unknown. Nevertheless, exactly how the dynamic process of declining renal function and metabolic syndrome are related. The study's purpose is to evaluate the causal relationship between MetS and the deterioration in kidney function using a Mendelian randomization (MR). Univariable and multivariable MR were applied to evaluate the causal relationships between MetS and its components with Rapid3, CKDi25, and CKD. The main source of MetS data was the GTC database, whose constituents came from extensive genome‐wide association research. The CKDGen Consortium provided data on dynamic changes in kidney function. Preliminary analysis was conducted using five different statistical techniques, including Inverse Variance Weighting and Weighted Median. Rucker's *Q*, MR‐Egger, and Cochran's *Q* test were used in sensitivity studies. In order to address reverse causality, the Steiger test was used. The IVW results showed Rapid3, CKDi25, and CKD all exhibited positive correlations with MetS. Rapid3, CKDi25, and CKD were found to have a positive causal relationship with SBP and WC, while DBP was also linked to an increased risk of Rapid3 and CKDi25. Even after accounting for other variables, MVMR analysis showed a correlation between WC and the drop in kidney function indices. MetS, together with its constituents WC, SBP, and DBP, are separate risk factors for the deterioration of renal function. However, the causal relationship between FBG, HDL, TG, and the decline in kidney function indicators remains uncertain.

## Graphical abstract

Mendelian randomization (MR) was used to assess the causal relationship between MetS and worsening renal function. The results showed that MetS and its components WC, SBP and DBP were independent risk factors for the deterioration of renal function. However, the causal relationship between FBG, HDL, TG and the decline of renal function indicators remains unclear.

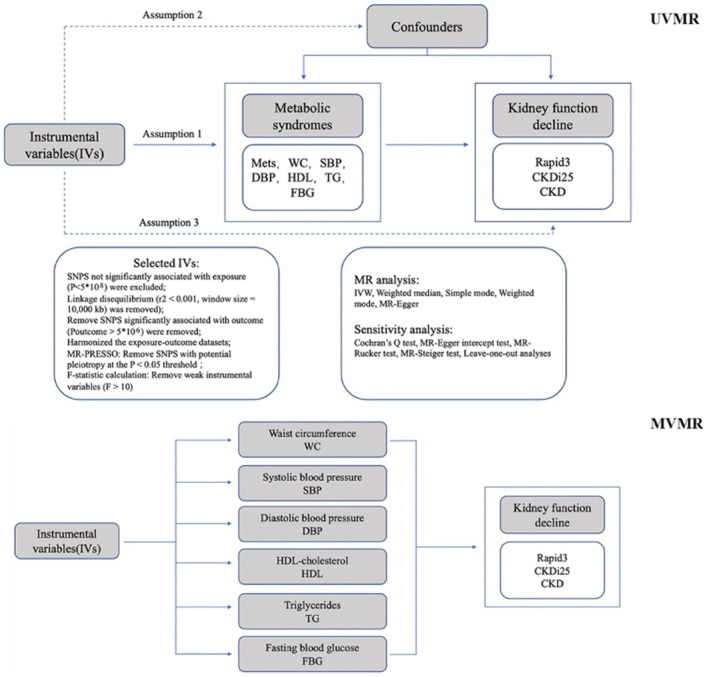

## 1 INTRODUCTION

One of the main causes of the global increase in morbidity and death is renal insufficiency, particularly chronic kidney disease.^1^ A collection of chronic conditions with glomerular filtration rate (GFR) < 60 mL/min/1.73 m^2^ are referred to as CKD and can be brought on by a number of primary kidney illnesses, diabetes, hypertension, and other kidney‐damaging factors.^2^, ^3^ The incidence of metabolic disorders such diabetes, obesity, hyperuricemia, and hypertension has steadily grown due to changes in lifestyle, aging populations, and improved living standards. This has led to an increase in the incidence of secondary CKD. Furthermore, an increasing body of research indicates that certain cardiovascular disease risk factors also influence the onset and progression of CKD, and these variables are inextricably linked to the development of metabolic syndrome.^4^, ^5^

Metabolic syndrome (MetS) is a clinical syndrome of systemic chronic inflammation and metabolic disorders caused by insulin resistance. A combination of metabolically linked risk factors, including central obesity, hypertension, improper glucose metabolism, and abnormal lipid metabolism, characterize MetS.^6^ The global incidence of MetS is rising quickly due to improvements in economic status and the spread of unhealthy lifestyles, particularly in emerging nations and areas.^7^, ^8^ Nevertheless, a thorough explanation of their causal connection has not yet been provided. The influence of MetS and its components on the risk of CKD was the subject of conflicting findings in cross‐sectional observational studies based on American and European populations.^9^, ^10^ According to other research, the MetS group had a greater prevalence of CKD than the overall population. In addition, CKD patients have a greater frequency of MetS and metabolic abnormalities than the general population.^11^, ^12^, ^13^

Genetic variations are used as instrumental variables in the MR statistical model.^14^ It is somewhat comparable to randomized controlled trials and can help prevent confounding, reverse causality, and other bias problems associated with observational research.^15^ The causal link between exposure variables and outcome factors may be assessed using this analytic approach.^16^ In order to provide a foundation for preventing and postponing the onset of renal impairment, MR Analysis was employed in this work to investigate the causal association between MetS and its components and renal function.

## 2 METHODS

### 2.1 Study design

**FIGURE 1:**
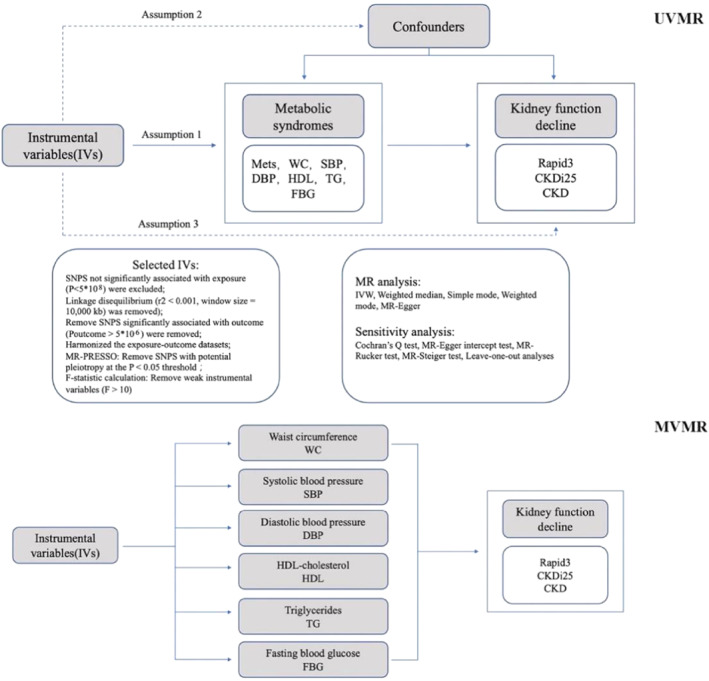
Flowchart of the current MR design.

To assess the causal effects of MetS and its related components on renal function using genetic variables, the two‐sample MR design required three key assumptions to be met: (1) the instrumental variables were associated with exposures; (2) The instrumental variables were not associated with any of the confounding factors affecting the association of exposure outcomes; and (3) Instrumental variables do not affect outcomes unless they are linked by exposure.^17^ Our study used only aggregate level data and therefore did not require ethical approval. The flowchart of this study is as follows (Figure 1).

For the selection of GWAS, we considered the following criteria:

(1) GWAS was conducted on individuals of European ancestry;
(2) The exposure and outcome datasets were unrelated to one another;
(3) The Global Data Analysis System summary data was acquired;
(4) A sizable sample size to provide sufficient statistical power;
(5) An ample supply of instrumental variables for MR Analysis.

### 2.2 GWAS information for MetS and its elements

We conducted a two‐sample MR Analysis after extracting IVs from the GWAS summary level datasets in order to investigate the possible causative link between MetS and renal function metrics. IVs found genetic variations of the metabolic syndrome using structural equation modeling techniques, based on new data from the Complex Trait Genetics Laboratory (CTG), which includes 461,920 valid participants of European ancestry.^18^ The IEU OpenGWAS database provided a string of data for the MetS component, allowing for statistical data download and systematic causal inference investigation.^19^, ^20^ Table 1 contains further details on exposure statistics.

**TABLE 1:** Overview of data sources.

Exposure	Ancestry	Sample size	Data sources	GWAS ID	Web source
Metabolic syndrome	European	461,920	CTG database	NA	https://ctg.cncr.nl/software/summary_statistics
WC	European	462,166	MRC‐IEU	Ukb‐b‐9405	https://gwas.mrcieu.ac.uk/datasets/ukb‐b‐9405/
SBP	European	436,419	MRC‐IEU	Ukb‐b‐20175	https://gwas.mrcieu.ac.uk/datasets/ukb‐b‐20175/
DBP	European	436,424	MRC‐IEU	Ukb‐b‐7992	https://gwas.mrcieu.ac.uk/datasets/ukb‐b‐7992/
HDL	Mixed (96% European)	187,167	GLGC	Ieu‐a‐299	https://gwas.mrcieu.ac.uk/datasets/ieu‐a‐299/
TG	Mixed (96% European)	177,861	GLGC	Ieu‐a‐302	https://gwas.mrcieu.ac.uk/datasets/ieu‐a‐302/
FBG	European	68,074	ALisaK Manning et al.	Ebi‐a‐GCST005186	https://gwas.mrcieu.ac.uk/datasets/ebi‐a‐GCST005186/

### 2.3 GWAS data on renal function

We looked for instrumental variables linked to CKD (the main outcome) using data from the CKDGen Consortium. The criteria for CKD was eGFR <60 mL/min/1.73 m^2^. A meta‐analysis of 23 European cohorts with 41,395 patients and 439,303 controls produced the GWAS data for CKD.^21^ Furthermore, we included two cohort studies as endpoints to assess the relationship between the metabolic syndrome and its constituent parts and dynamic changes in renal function. Rapid decline of kidney function (Rapid3) (34,874 cases and 107,090 controls) and rapid progress To CKD (CKDi25) (19,901 cases and 175,244 controls) from the CKDGen consortium and a GWAS meta‐analysis of 42 European ancestry studies conducted primarily in the UK Biobank.^22^ A decline in eGFR of more than 3 mL/min/1.73 m^2^ per year was referred to as Rapid3, and a decline in eGFR of more than 25% and the transition from no CKD to CKD was referred to as CKDi25.

### 2.4 Selection of genetic tools for MetS associated phenotypes

We selected the genetic tools based on the following criteria:

a) SNPs significant over the whole genome (*P* < 5 × 10^8^);
b) Removal of linkage disequilibrium (*r* ^2^ threshold = 0.001 and clumping distance = 10,000 kb);
c) We extracted SNPS from GWAS outcome (renal function) data. In this step, SNPS associated with genomewide significant outcomes (*p* < 5 × 10^6^) were removed, and SNPS missing from outcomes were chosen to be discarded;
d) We excluded ambiguous and palindromic SNPS (minor allele frequency >0.42) with incorrect effects in the coordination process;
e) The F‐statistic was used to ensure a strong correlation between IVs and exposure. An F‐statistic greater than 10 is generally considered to meet the criteria for a strong correlation.

These screening requirements guarantee the validity of this study's findings.

### 2.5 Two‐sample MR main analysis

The random‐effects IVW approach was the main statistical technique employed in this study's UVMR analysis to determine the possible causal link between MetS and renal function. Because it may yield a steady outcome even in the absence of horizontal pleiotropy, this approach was selected.^23^ The IVW findings, however, will be significantly skewed if the instrumental factors are not entirely independent of one another.^24^ As a result, we decided to include the MR‐Egger method, weighted mode, weighted median estimate, and simple model as extra techniques. The IVW technique's condition that there be no horizontal pleiotropy among genetic variations is relaxed by the MR‐Egger approach, which is used to identify and compensate for horizontal pleiotropy in MR analysis.^25^ When at least 50% of the data originates from reliable instrumental variables, weighted median and weighted mode can produce efficient and reliable analytical findings.^26^

### 2.6 Sensitivity analysis

For significant results (IVW: *p* < 0.05), we employed multiple methods for quality control. Firstly, we used Cochran's *Q* test to assess the heterogeneity of the IVW model, where a Cochran *Q* test with *p* < 0.05 and *I* ^2^ > 25% was considered indicative of heterogeneity.^27^ However, the presence of heterogeneity does not necessarily invalidate the random‐effects IVW estimation when overall heterogeneity is balanced. Rucker's *Q* test was used to evaluate the heterogeneity of the MR‐Egger regression, and differential Q‐Q′ was obtained to assess the suitability of IVW for interpreting specific causal inferences.^28^ A small difference in Q‐Q' (*p* > 0.05) indicated good fit for the IVW model. Additionally, we evaluated horizontal pleiotropy based on the intercept term derived from the MR‐Egger regression. The Leave‐one‐out (LOO) analysis method was employed to assess whether individual SNPs influenced the main causal association. We applied MR Steiger filtering to determine the direction of the causal relationship between each instrumental variable and the exposure and outcome. The Steiger filtering method assumes that an effective instrument should explain more exposure variance than outcome variance and classifies the instrument's direction as “TRUE” if it meets the standard, otherwise as “FALSE”.^29^ To evaluate the validity and reliability of the study results, we conducted statistical power calculations (https://shiny.cnsgenomics.com/mRnd/).^30^

### 2.7 Multivariate Mendelian randomization analysis

To confirm the direct causative associations between MetS and its components with Rapid3, CKDi25, and CKD, multivariable Mendelian randomization (MVMR) was applied. Using the mvm‐ivw, mvm‐egger, mvm‐lasso, and mvm‐median approaches, the direct causality was established.^31^ It is deemed that the causal association endures even after multivariable correction if at least one of these five techniques produces statistically significant findings (Figure 1).

### 2.8 Statistical analysis

We used a Bonferroni‐corrected threshold of *p* < 0.003 (*α* = 0.05/18) as the criteria for statistical significance (considered significant) because of the multiple testing in the initial analysis. Since Bonferroni correction is a conservative technique, *p*‐value estimates in this study that fell between 0.05 and 0.003 were deemed to have nominal significance. The software packages TwoSampleMR, MR‐PRESSO, Mendelian Randomization, and MVMR in R (version 4.10) were used for all of the studies.

## 3 RESULTS

### 3.1 Genetic instruments selected in MR

The study selected phenotypes related to MetS and its 6 associated components to analyze the causal effects on the pathological conditions of three states of renal function decline, namely Rapid3, CKDi25, and CKD. After rigorous screening, the number of participants used for each phenotype varied from 12 to 319. F‐statistics for SNPs were all over 10, suggesting that bias due to the use of weak instrumental variables was unlikely. The final list of retained SNPs is presented in Supplementary Tables S1–S21. The statistical power are displayed in Table S22.

### 3.2 Genetic prediction of the relationship between MetS and renal function decline

**FIGURE 2:**
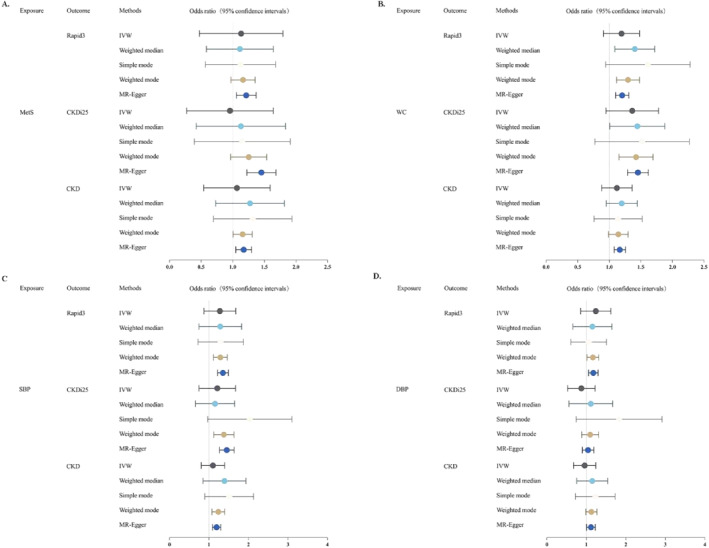
Odds ratio for association of genetically predicted MetS and its components with renal function decline indexes.

The results of the MR analysis on the association between MetS and Rapid3, CKDi25, and CKD are shown in Figure 2, Table 2 and Supplementary Figure S2. The causal relationship between MetS and renal function decline demonstrates different characteristics under different physiological states. Evidence provided by the IVW analysis indicates a positive causal relationship between MetS and Rapid3, CKDi25, and CKD. The specific data are as follows: the OR for Rapid3 is 1.17 (95% CI = 1.05–1.30, *P* = 3.53E‐03); the OR for CKDi25 is 1.44 (95% CI = 1.23–1.69, *P* = 7.79E‐06); and the OR for CKD is 1.21 (95% CI = 1.06–1.37, *P* = 3.45E‐03). Among these, the association between MetS and CKDi25 is statistically significant (*p* < 0.003), while the association with Rapid3 and CKD reaches nominal significance (*p* < 0.05). Through the Cochran *Q* test, it was found that there is no significant heterogeneity between MetS and Rapid3 and CKDi25, and there is no significant level of pleiotropy. For the causal relationship between MetS and CKD, the Cochran *Q* test indicates the presence of heterogeneity but not significant (Table 3). However, the slight Q‐Q′ difference suggests that IVW may be an appropriate causal inference model. Leave‐one‐out analysis indicates that the estimated effect is not influenced by specific SNPs (Supplementary Figure S1). Steiger filtering also did not reveal any reverse causal SNPs, indicating the reliability of this causal relationship direction (Table 3).

**TABLE 2:** Association of MetS and its components with renal function decline in MR analysis.

Exposure	Estimates mtehod	Rapid3	*p*	CKDi25	*p*	CKD	*p*
		OR (95% CI)		OR (95% CI)		OR (95% CI)	
MetS	IVW	1.17 (1.05, 1.30)	0.0035	1.44 (1.23, 1.69)	7.79E‐06	1.21 (1.06.1.37)	0.0035
	Weighted median	1.17 (1.0, 1.30)	0.0489	1.23 (0.98, 1.55)	0.0799	1.15 (0.98, 1.36)	0.0862
	Simple mode	1.22 (0.75, 1.98)	0.4182	0.98 (0.49, 1.98)	0.9543	1.03 (0.62, 1.72)	0.9078
	Weighted mode	1.20 (0.77, 1.85)	0.4236	0.98 (0.51, 1.9)	0.9518	1.03 (0.63, 1.68)	0.9038
	MR‐Egger	0.98 (0.59, 1.63)	0.9457	0.79 (0.37, 1.71)	0.5536	1.00 (0.55, 1.85)	0.9876
WC	IVW	1.16 (1.08, 1.26)	1.47E‐04	1.45 (1.29, 1.62)	1.01E‐10	1.20 (1.10, 1.31)	2.79E‐05
	Weighted median	1.14 (0.99, 1.30)	0.6078	1.41 (1.16, 1.70)	4.72E‐04	1.29 (1.12, 1.48)	2.98E‐04
	Simple mode	1.10 (0.78, 1.54)	0.5852	1.40 (0.84, 2.32)	0.1984	1.52 (0.99, 2.32)	0.0554
	Weighted mode	1.18 (0.96, 1.45)	0.1137	1.40 (1.03, 1.90)	0.0336	1.38 (1.10, 1.73)	0.0055
	MR‐Egger	1.10 (0.89, 1.37)	0.3759	1.32 (0.97, 1.80)	0.0774	1.17 (0.92, 1.49)	0.1921
SBP	IVW	1.19 (1.09, 1.30)	1.06E‐04	1.44 (1.27, 1.64)	3.00E‐08	1.35 (1.22, 1.49)	1.32E‐08
	Weighted median	1.23 (1.08, 1.40)	0.0019	1.36 (1.13, 1.64)	0.0013	1.28 (1.12, 1.47)	3.62E‐04
	Simple mode	1.43 (0.94, 2.17)	0.0969	1.85 (1.07, 3.18)	0.023	1.21 (0.77, 1.91)	0.4053
	Weighted mode	1.32 (0.89, 1.97)	0.164	1.08 (0.70, 1.68)	0.728	1.21 (0.79, 1.86)	0.3799
	MR‐Egger	1.07 (0.82, 1.41)	0.6192	1.15 (0.78, 1.70)	0.4743	1.23 (0.90, 1.70)	0.2011
DBP	IVW	1.11 (1.01.1.23)	0.029	1.04 (0.90, 1.19)	0.6197	1.17 (1.06, 1.30)	2.06E‐03
	Weighted median	1.12 (0.99.1.27)	0.0759	1.08 (0.89, 1.32)	0.4363	1.16 (1.02, 1.32)	0.0262
	Simple mode	1.16 (0.76, 1.76)	0.5002	1.61 (0.87, 3.01)	0.1325	1.00 (0.64, 1.54)	0.9816
	Weighted mode	1.11 (0.78, 1.56)	0.5686	1.02 (0.61, 1.71)	0.9432	1.08 (0.70, 1.68)	0.7189
	MR‐Egger	0.93 (0.69, 1.25)	0.6268	0.83 (0.55, 1.24)	0.3592	1.20 (0.88.1.64)	0.2563
HDL	IVW	0.96 (0.91, 1.02)	0.1724	0.91 (0.84.0.98)	0.0178	0.97 (0.91, 1.03)	0.3318
	Weighted median	1.01 (0.93, 1.09)	0.9033	0.96 (0.86, 1.09)	0.5409	0.97 (0.89, 1.05)	0.4458
	Simple mode	0.98 (0.85, 1.12)	0.7407	0.93 (0.75, 1.15)	0.5028	1.00 (0.83, 1.20)	0.9987
	Weighted mode	1.00 (0.92, 1.08)	0.9964	0.96 (0.85, 1.09)	0.5386	1.01 (0.94, 1.10)	0.7516
	MR‐Egger	1.05 (0.94, 1.17)	0.3735	1.08 (0.94.1.26)	0.2829	1.06 (0.94, 1.20)	0.3522
TG	IVW	1.00 (0.94, 1.07)	0.9049	1.07 (0.98, 1.16)	0.163	1.05 (0.97, 1.14)	0.2325
	Weighted median	0.97 (0.89, 1.06)	0.5289	1.05 (0.94, 1.18)	0.3956	1.01 (0.92, 1.11)	0.813
	Simple mode	1.05 (0.90, 1.22)	0.5805	1.10 (0.89, 1.35)	0.3799	1.01 (0.85.1.20)	0.9536
	Weighted mode	1.01 (0.92, 1.11)	0.8047	1.08 (0.97, 1.20)	0.1833	1.03 (0.94, 1.12)	0.5421
	MR‐Egger	1.00 (0.90, 1.11)	0.9891	1.06 (0.92, 1.22)	0.4268	0.97 (0.85, 1.11)	0.6717
FBG	IVW	1.02 (0.86, 1.22)	0.7823	1.08 (0.80, 1.44)	0.624	0.98 (0.79, 1.22)	0.8877
	Weighted median	1.03 (0.83.1.29)	0.7699	1.00 (0.74, 1.34)	0.9934	0.93 (0.75, 1.16)	0.5383
	Simple mode	1.20 (0.85, 1.69)	0.3168	1.06 (0.67, 1.67)	0.8115	1.22 (0.84, 1.77)	0.3211
	Weighted mode	1.02 (0.80, 1.29)	0.903	1.01 (0.74, 1.37)	0.9713	0.97 (0.78, 1.20)	0.7682
	MR‐Egger	0.90 (0.61, 1.32)	0.6087	1.01 (0.51, 1.99)	0.9782	1.16 (0.72, 1.87)	0.5479

**TABLE 3:** Sensitivity analysis for the causal association between MetS and its components and renal function decline.

Exposure	Ourcome	Cochram			MR‐Egger		Q‐Q′		Steiger direction	
		IVW Q	*p*	*I* ^2^	Intercept	*p*	*Q*	*p*	correct_direction	steiger_pval
MetS	Rapid3	181.709	0.5546368	0%	0.0025661	0.49	0.475326	0.490547	TRUE	6.54E‐209
	CKDi25	201.957	0.2	7.90%	0.0087897	0.12	2.627461	0.10503	TRUE	0
	CKD	267.153	7.29E‐05	30.70%	0.0027143	0.55	0.529674	0.466744	TRUE	0
WC	Rapid3	341.482	0.17	0.072	0.0008801	0.61	0.27846	0.597713	TRUE	0
	CKDi25	330.673	0.29	4.10%	0.0015137	0.54	0.392126	0.531184	TRUE	0
	CKD	420.193	1.03E‐04	2.40%	0.0003973	0.84	0.057461	0.810555	TRUE	0
SBP	Rapid3	251.906	0.05	14.30%	0.0019854	0.43	0.729302	0.39311	TRUE	0
	CKDi25	250.45	0.07	13%	0.0042402	0.24	1.612317	0.204166	TRUE	0
	CKD	338.831	1.12E‐07	37%	0.0016899	0.57	0.522208	0.469902	TRUE	0
DBP	Rapid3	293.504	2.50E‐04	27.10%	0.004352558	0.25	2.20638	0.137441	TRUE	0
	CKDi25	280.931	2.59E‐03	22%	0.0043526	0.21	1.699555	0.192346	TRUE	0
	CKD	326.411	9.06E‐07	35%	−0.0004096	0.87	0.031302	0.859567	TRUE	0
HDL	Rapid3	106.455	0.05	21%	−0.0047211	0.06	4.540784	0.033096	TRUE	0
	CKDi25	98.095	0.16	13%	−0.0092408	0.01	8.123469	0.00437	TRUE	0
	CKD	128.105	5.16E‐04	38%	−0.0048973	0.09	4.665747	0.03077	TRUE	0
TG	Rapid3	69.105	0.08	21.90%	0.000201	0.94	0.007982	0.92881	TRUE	0
	CKDi25	63.323	0.18	14.70%	0.0002938	0.94	0.008083	0.928361	TRUE	0
	CKD	94.85	1.88E‐04	46.20%	0.0047791	0.13	4.20379	0.040334	TRUE	0
FBG	Rapid3	13.936	0.31	13.90%	0.005275	0.48	0.642421	0.422836	TRUE	7.48E‐250
	CKDi25	20.006	0.07	40%	0.0026592	0.84	0.0767	0.78182	TRUE	2.48E‐270
	CKD	18.895	0.06	41.80%	−0.0069105	0.46	1.078243	0.299091	TRUE	9.11E‐305

### 3.3 Association of genetically predicted components of MetS syndrome with renal function states

After Bonferroni correction, the IVW results of our primary MR analysis revealed significant associations between waist circumference (WC) (Rapid3: OR = 1.16, 95% CI = 1.08–1.23, *P* = 1.47E‐04; CKDi25: OR = 1.45, 95% CI = 1.29–1.62, *P* = 1.01E‐10; CKD: OR = 1.20, 95% CI = 1.10–1.31, *P* = 2.79E‐05) and systolic blood pressure (SBP) (Rapid3: OR = 1.19, 95% CI = 1.09–1.30, *P* = 1.06E‐04; CKDi25: OR = 1.44, 95% CI = 1.27–1.64, *P* = 3.00E‐08; CKD: OR = 1.35, 95% CI = 1.22–1.49, *P* = 1.32E‐08) with an increased risk of Rapid3, CKDi25, and CKD. Diastolic blood pressure (DBP) (Rapid3: OR = 1.11, 95% CI = 1.11–1.23, *p* = 0.029; CKD: OR = 1.17, 95% CI = 1.06–1.30, *P* = 2.06E‐03) showed detrimental effects on Rapid3 and CKD (Figure 2, Table 2 and Supplementary Figure S2). Among these, the association with CKD was significant, while the association with Rapid3 displayed nominal significance. Our estimates indicated that high‐density lipoprotein (HDL), triglycerides (TG), and fasting blood glucose (FBG) did not show statistical significance in the progression of renal function status (Table 2) (we noted that although the initial IVW results for HDL and CKDi25 had a *p*‐value less than 0.05, the Q‐Q′ plot indicated that the IVW analysis results were unreliable, and thus we did not consider them in the positive results). We performed a number of sensitivity studies on these positive results to confirm the accuracy of our MR analysis and prevent undue bias. The intercept of the MR Egger regression did not exhibit directional pleiotropy (*p* > 0.05). And except for a few exposure‐outcome causal links (Table 3), the Cochran's *Q* test indicated no significant heterogeneity present. As demonstrated by leave‐one‐out analysis (Supplementary Figure S1), the estimated effects were not found to be reliant on any particular SNP. Similarly, the Rucker test demonstrated that the minor Q‐Q' difference suggested a strong match for the IVW model (Table 3). Through Steiger's test, we confirmed that all the MR estimates were not influenced by reverse causation (Table 3).

### 3.4 Multivariable Mendelian randomization

To better understand the complex interactions between these identified factors, we conducted a MVMR analysis, comprehensively assessing their associations with renal function decline (Table 4). In the MVMR analysis, WC showed an association with the increased risk of Rapid3 (IVW: OR = 1.15, 95% CI = 1.04–1.28, *p* = 0.008) and CKDi25 (IVW: OR = 1.43, 95% CI = 1.23–1.67, *P* = 4.13E‐06), although the IVW results indicated a less significant association between WC and CKD. However, Lasso analysis revealed a detrimental impact of WC on CKD (Lasso: OR = 1.14, 95% CI = 1.03–1.27, *p* = 0.015). Systolic blood pressure showed a significantly positive correlation with the risk of CKDi25 (IVW: OR = 1.64, 95% CI = 1.28–2.10, *P* = 1.01E‐04), and DBP displayed a significantly positive correlation with the risk of CKD (IVW: OR = 1.29, 95% CI = 1.07–1.56, *p* = 0.0075). However, the causal effects of SBP on Rapid3 and CKD, and DBP on Rapid3, were no longer evident. This discrepancy from the analysis results of UVMR could possibly be explained by the mediation of other components of MetS in their causal effects on the indicators of renal function decline.

**TABLE 4:** The associations between MetS and its components and renal function decline according to multivariable Mendelian randomization analysis.

Exposure	Method	Rapid3		CKDi25		CKD	
		OR (95% CI)	*p*‐value	OR (95% CI)	*p*‐value	OR (95% CI)	*p*‐value
WC	IVW	1.1537 (1.0380, 1.2822)	8.00E‐03	1.4312 (1.2287, 1.6671)	4.13E‐06	1.1041 (0.9754, 1.2499)	0.1175
	Lasso	1.1852 (1.0708, 1.3119)	8.20E‐03	1.3888 (1.1989, 1.6087)	1.19E‐05	1.1413 (1.0259, 1.2698)	1.51E‐02
	Egger	1.1553 (1.0381, 1.2858)	8.20E‐03	1.4289 (1.2241, 1.6679)	6.12E‐06	1.1011 (0.9714, 1.2482)	0.1321
SBP	IVW	1.1462 (0.9678, 1.3575)	0.1139	1.6417 (1.2786, 2.1081)	1.01E‐04	1.0538 (0.8619, 1.2884)	0.6095
	Lasso	1.1138 (0.9464, 1.3107)	0.1946	1.6988 (1.3342, 2.1629)	1.71E‐05	1.0400 (0.8749, 1.2262)	0.6564
	Egger	1.1473 (0.9682, 1.3596)	0.1127	1.6397 (1.2757, 2.1078)	1.13E‐04	1.0515 (0.8593, 1.2866)	0.626
DBP	IVW	0.9792 (0.8360, 1.1469)	0.7942	0.7899 (0.6245, 0.9989)	0.05	1.2918 (1.0707, 1.5584)	7.50E‐03
	Lasso	1.0061 (0.8642, 1.1714)	0.9371	0.8105 (0.6466, 1.0161)	0.0685	1.3360 (1.1348, 1.5730)	5.00E‐04
	Egger	0.9793 (0.8359, 1.1472)	0.7952	0.7899 (0.6243, 0.9993)	0.05	1.2910 (1.0697, 1.5580)	7.80E‐03
HDL	IVW	0.9610 (0.9010, 1.0251)	0.2273	0.9509 (0.8657.1.0446)	0.2938	0.9740 (0.9026, 1.0512)	0.4985
	Lasso	0.9672 (0.9092, 1.0289)	0.29	0.9492 (0.8672, 1.0390)	0.25841	1.0097 (0.9478, 1.0757)	0.7652
	Egger	0.9607 (0.9005, 1.0250)	0.225	0.9512 (0.8657, 1.0452)	0.2983	0.9746 (0.9029, 1.0520)	0.5092
TG	IVW	0.9771 (0.9142, 1.0445)	0.4964	1.0535 (0.9568, 1.1600)	0.2885	0.9722 (0.8986, 1.0519)	0.4831
	Lasso	0.9834 (0.9226, 1.0482)	0.607	1.0533 (0.9598, 1.1549)	0.2918	1.0422 (0.9711, 1.1184)	0.2515
	Egger	0.9774 (0.9143, 1.0449)	0.5027	1.0529 (0.9564, 1.1599)	0.2918	0.9717 (0.8979, 1.0515)	0.4757
FBG	IVW	1.0486 (0.8978, 1.2248)	0.5491	0.9785 (0.7821, 1.2242)	0.8491	1.1154 (0.9271, 1.3419)	0.2471
	Lasso	1.0595 (0.9125.1.2303)	0.4481	0.9880 (0.7964, 1.2257)	0.9126	1.0630 (0.9092, 1.2428)	0.4438
	Egger	1.0572 (0.8840, 1.2643)	0.5425	0.9702 (0.7504, 1.2545)	0.8177	1.0972 (0.8867, 1.3577)	0.3932

## 4 DISCUSSION

The connection between MetS and CKD has garnered a lot of attention lately.^32^, ^33^ Even though there is a large body of research examining the connection between these two conditions, the majority of studies have been observational and have a clinical background. This can introduce biases and confounding variables, which can skew the intricate causal relationships between variables. Therefore, our study used MR approaches, such as UVMR and MVMR, for in‐depth examination in order to thoroughly evaluate the causal association between MetS and its components with the progression of CKD.

Based on the results of UVMR analysis, we found a causal relationship between MetS and Rapid3, CKDi25, and CKD, which exhibited strong associations with CKDi25 and nominal significance with Rapid3 and CKD. Furthermore, we conducted MVMR analysis to further investigate the components of MetS. The results showed that after adjusting for related exposures, no independent effects of systolic blood pressure on Rapid3 and CKD, as well as diastolic blood pressure on Rapid3, were observed, while only WC remained associated with the three indicators of renal function decline (Rapid3, CKDi25, and CKD).

The kidneys are highly vascularized organs and are prone to microvascular changes. MetS, characterized by inflammation and increased oxidative stress, leads to pathological changes such as endothelial dysfunction and a prothrombotic state, accelerating kidney damage. Therefore, MetS and its components are related to the onset and development of CKD. Numerous studies have found a strong correlation between metabolic syndrome and the incidence of chronic kidney disease. Reports by Navanethan et al. indicate a clear correlation between MetS and the progression of CKD to end‐stage renal disease (ESRD).^34^ Similarly, studies by Watanabe et al. found that MetS increased the risk of ESRD and death, demonstrating that MetS is an important factor associated with the risk of CKD.^35^ This evidence aligns with our research findings.

WC is acknowledged as the best measurement of visceral fat mass, showing a linear relationship with abdominal obesity and serving as a reliable indicator of central obesity. Obesity is considered to be associated with an increased incidence of CKD and is a fundamental component of MetS.^36^ Obesity increases renal reabsorption of sodium and water, leading to increased extracellular matrix, resulting in glomerulosclerosis and glomerular hypertrophy, leading to conditions such as hypertension and glomerular hyperfiltration, activating the renin‐angiotensin‐aldosterone system (RAAS) and the sympathetic nervous system, ultimately resulting in hypertension and kidney damage.^37^ However, obesity leads to a range of metabolic disturbances, including insulin resistance, diabetes, hypertension, and abnormal lipid metabolism. This raises the question that frequently troubles researchers: does obesity cause kidney damage, or does it result from other metabolic abnormalities caused by obesity? Therefore, we utilized the MVMR method to determine the independent causal relationship between waist circumference and Rapid3, CKDi25, and CKD, which revealed that the influence of WC on these variables may be independent and not subject to interference from other factors.

Hypertension can cause multi‐organ dysfunction, such as in the heart, brain, arteries, eyes, and kidneys, and significantly increase the risk of cardiovascular events. In hypertensive patients, if blood pressure is not well controlled, some patients may develop proteinuria, and in later stages, a decline in eGFR may occur. Research by Kurella et al. indicates that in comparison to other components of MetS, hypertension plays a decisive role in the development of CKD.^9^ Other studies also suggest that the correlation of SBP with kidney outcomes may be stronger than that of DBP.^38^ Although previously, the UVMR analysis results showed a causative relationship between increased blood pressure and the risk of renal function decline, MVMR analysis demonstrated that the impact of hypertension on renal function may yet be influenced by other factors, necessitating further investigation of the underlying complex mechanisms in future studies.

While some studies have found that blood glucose and lipid metabolism have a close relationship with CKD, the exact impact of these factors on the onset and progression of CKD, and their correlation to the risk of developing renal insufficiency, remain controversial.^4^, ^39^ For individuals with MetS, an elevated FBG level is defined as ≥100 mg/dL (5.6 mmol/L).^40^ Most individuals at this level do not meet the diagnostic criteria for diabetes (fasting blood glucose >7 mmol/L) and may be in a state of impaired glucose tolerance. In this state, the kidneys are in a state of hyperfiltration, and kidney function does not show significant impairment. This might explain why the causative relationship between FBG and the outcome of this study—renal function decline—is not clear. Differences in the results of MR studies may be due to variations in the sources and sample sizes of the selected study outcome factors. Therefore, expanding the research sample and further exploring the causal relationship between blood glucose and lipid levels and kidney function could provide valuable insights.

Many historical studies have adopted a retrospective design, inevitably introducing unavoidable confounding factors. The significant financial investment and lengthy duration associated with randomized controlled trials create considerable pressure for researchers. In this context, our study has several clear advantages: Firstly, we used MR design methods, effectively avoiding potential confounding and reverse causal error. Secondly, we utilized genome‐wide association study (GWAS) summary data to investigate MetS and its components, enabling higher statistical power. Thirdly, we incorporated various sensitivity analyses to enhance the reliability of the study results. However, we must acknowledge the limitations of this study: for example, the lack of GWAS data for Asian populations, which needs consideration when extrapolating study findings to local environments. Additionally, due to limitations in genomic association study databases, we were unable to obtain more detailed information, such as ethnicity or disease severity.

## 5 CONCLUSION

Our data provide genetic evidence supporting a possible causal relationship between MetS and CKD progression. Metabolic syndrome and its components, waist circumference, systolic blood pressure and diastolic blood pressure may be independent risk factors for renal function deterioration.

## AUTHOR CONTRIBUTIONS

Deying Zhang and Qitong Guo conceived of the presented idea, developed the theory and performed the computations; Meiling Chen, Yihang Yu, Ping Li and Xu Huang wrote the main manuscript text; Chunlan Long and Lianju Shen conducted the MR analyses and prepared the graphs and tables; Xing Liu and Guanghui Wei verified the analytical methods. All authors have read and agreed to the published version of the manuscript.

## CONFLICT OF INTEREST STATEMENT

The authors declare that they have no competing interests.

## ETHICS STATEMENT

Our analysis used publicly available genome‐wide association study (GWAS) summary statistics. No new data were collected, and no new ethical approval was required.

## CONSENT FOR PUBLICATION

All authors approved the final manuscript and the submission to this journal.

## Supporting information

Supporting Information S1

Supporting Information S2

## Data Availability

The data that support the findings of this study are available from the corresponding author upon reasonable request.
