# Supplementary material for: Causal association of metabolic syndrome with chronic kidney disease progression: A Mendelian randomization study
Source: Pediatr Discov. 2024 Jul 6;2(4):e93. doi: 10.1002/pdi3.93 (PMC12118223; doi:10.1002/pdi3.93)
Supplement: Supplementary file 1 — Supporting Information S1 [file PDI3-2-e93-s001.docx]

Figure S1. Leave-one-out analysis for the Mendelian randomization (MR) association (P < 0.05) between a specific exposure and renal function decline index. Within each panel, the black points represent the causal estimate of association between a specific exposure and renal function decline index after discarding each SNP in turn. Red points represent the overall causal estimate using the random-effects inverse variance weighted. Horizontal lines denote 95% confidence intervals.


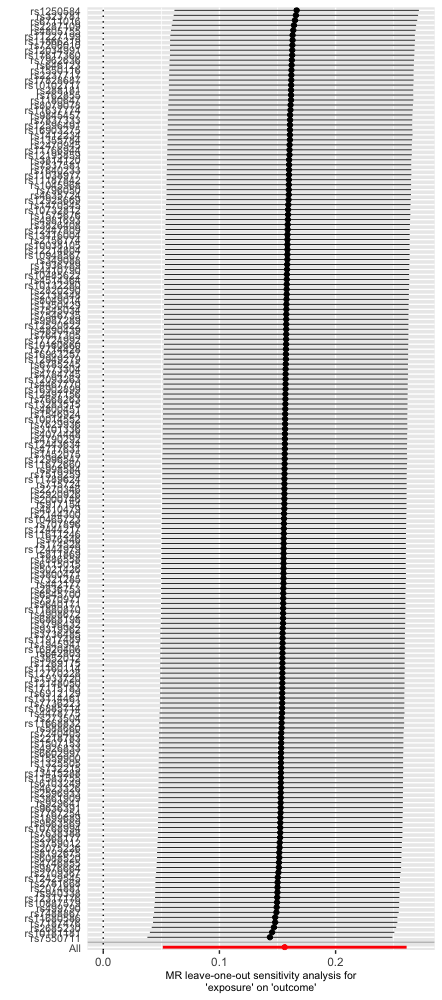


1. MR leave-one-out sensitivity analysis for Mets on Rapid3


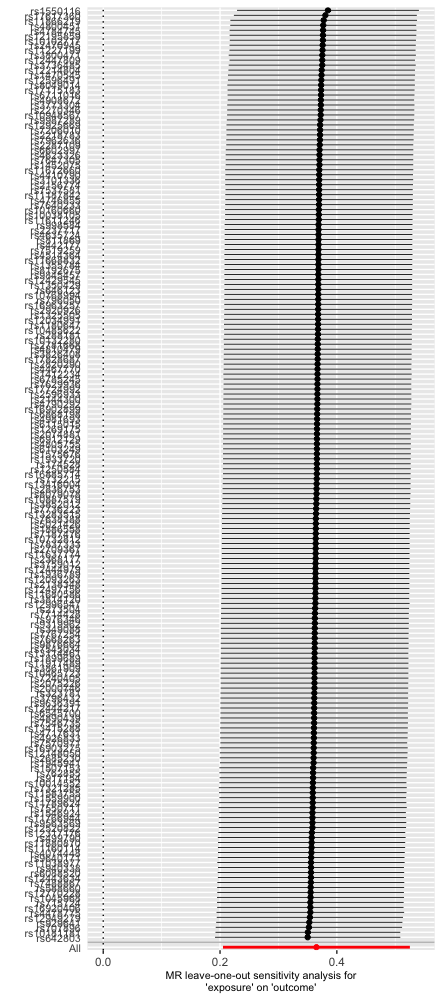


1. MR leave-one-out sensitivity analysis for Mets on CKDi25


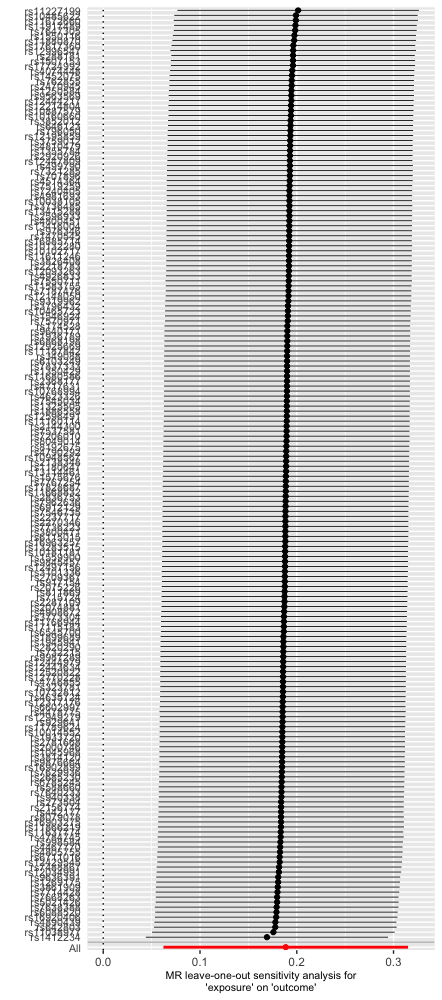


1. MR leave-one-out sensitivity analysis for Mets on CKD


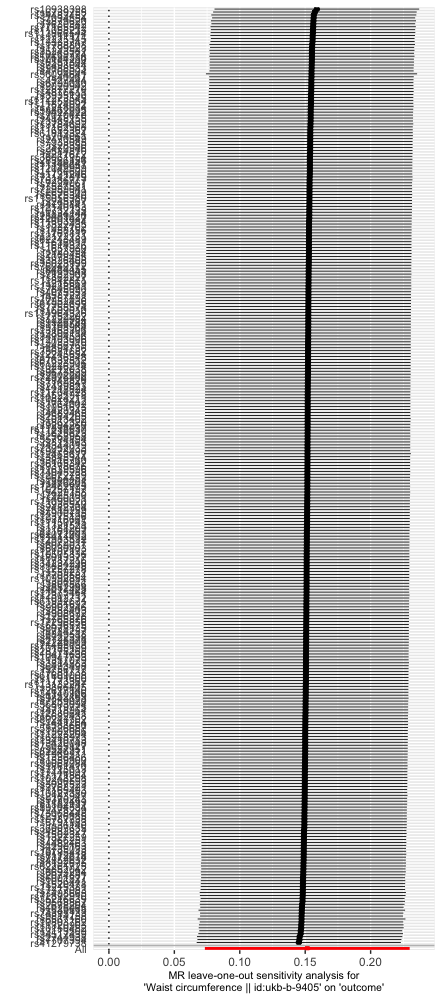


1. MR leave-one-out sensitivity analysis for WC on Rapid3


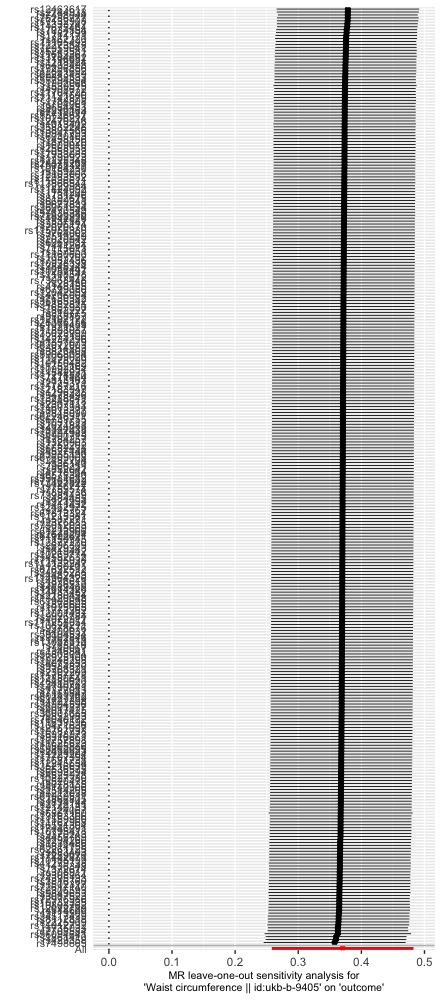


1. MR leave-one-out sensitivity analysis for WC on CKDi25


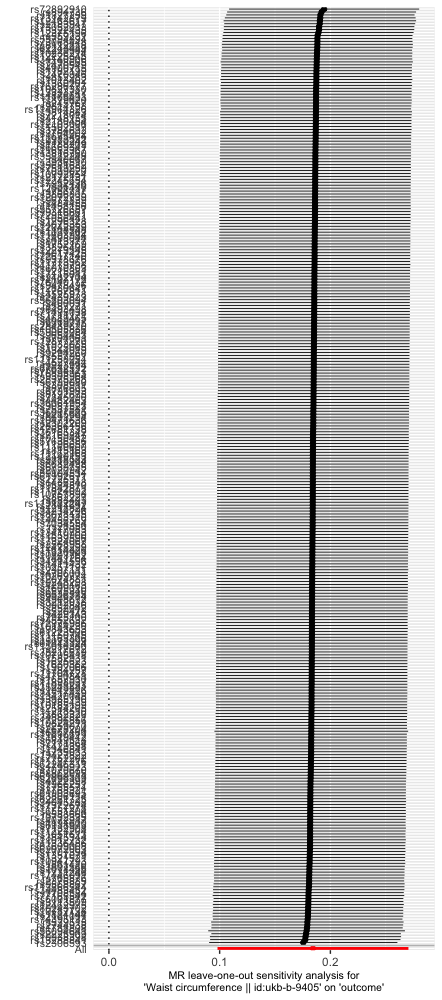


1. MR leave-one-out sensitivity analysis for WC on CKD


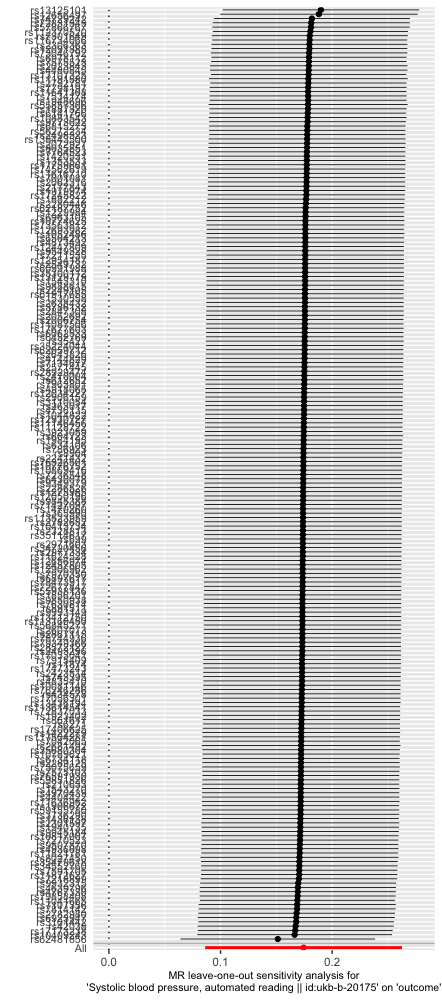


1. MR leave-one-out sensitivity analysis for SBP on Rapid3


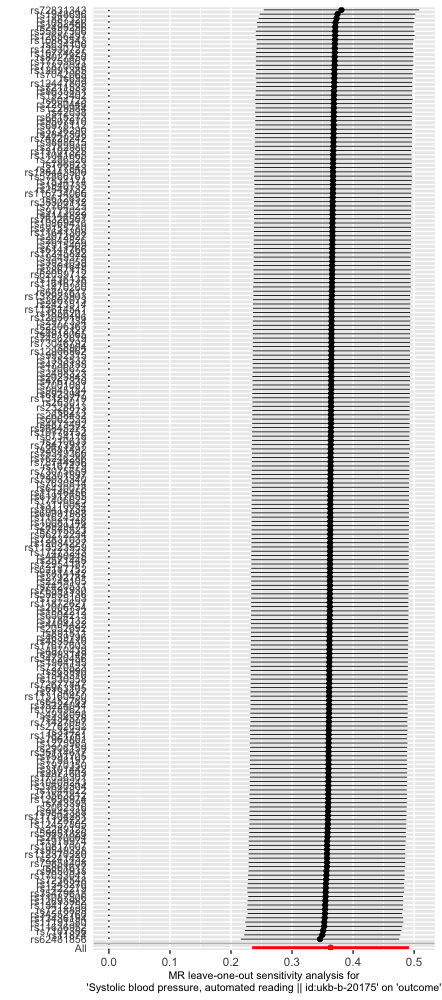


1. MR leave-one-out sensitivity analysis for SBP on CKDi25


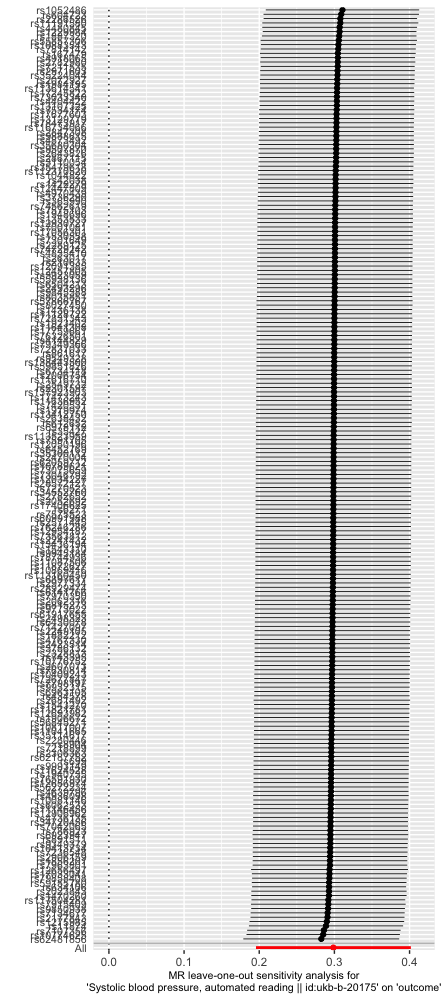


1. MR leave-one-out sensitivity analysis for SBP on CKD


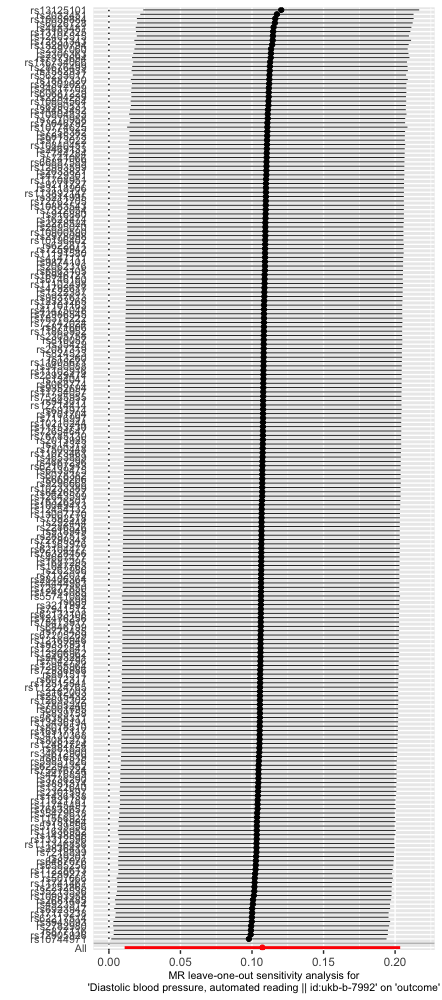


1. MR leave-one-out sensitivity analysis for DBP on Rapid3


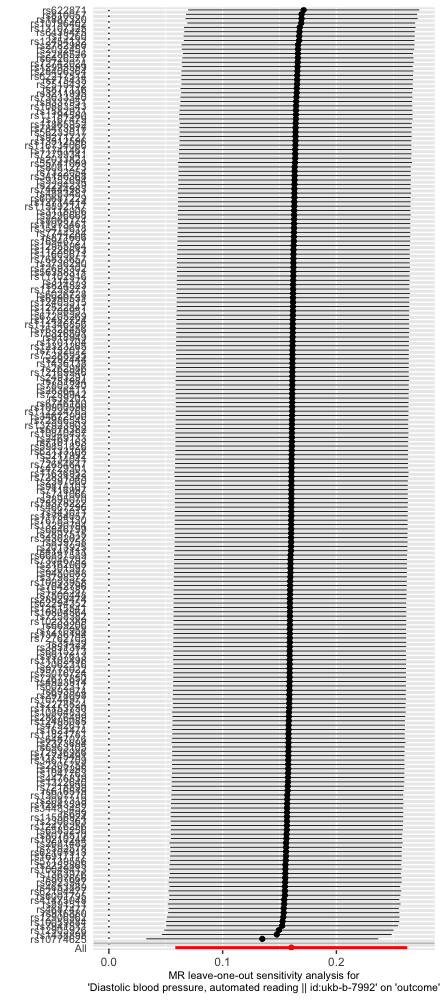


1. MR leave-one-out sensitivity analysis for DBP on CKD

Figure.2 Scatterplot for the Mendelian randomization (MR) association (P < 0.05) between a specific exposure and renal function decline index.

A
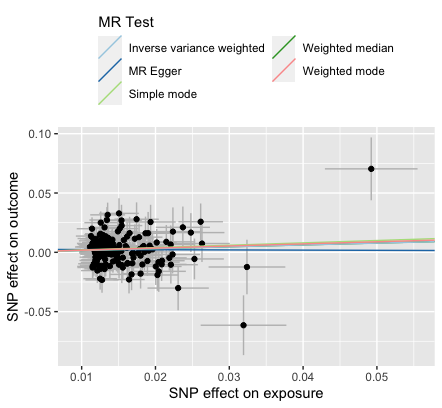


B
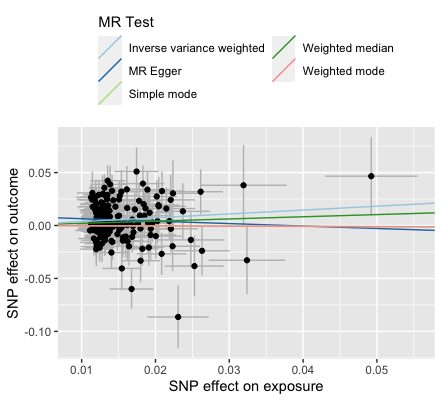


C
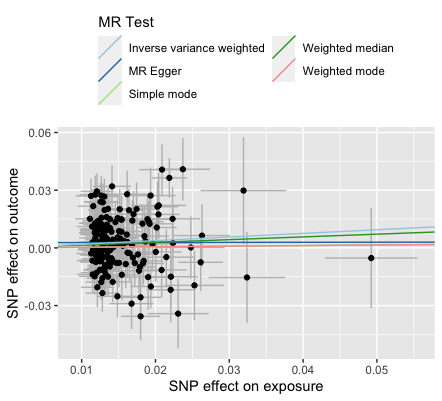


Scatterplot of Mendelian randomization (MR) estimates of genetic risk of Mets on Rapid3(A), CKDi25(B) and CKD(C).

D
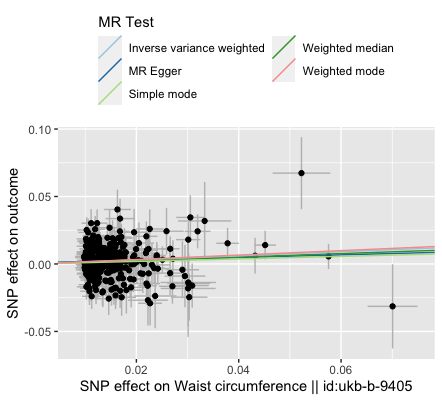


E
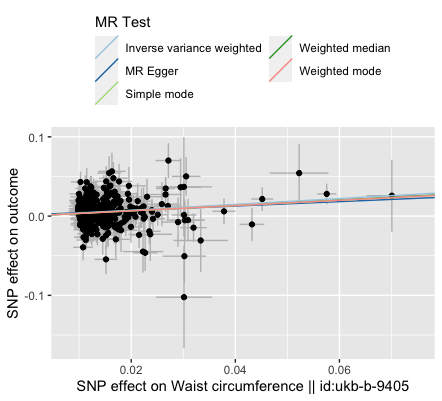


F
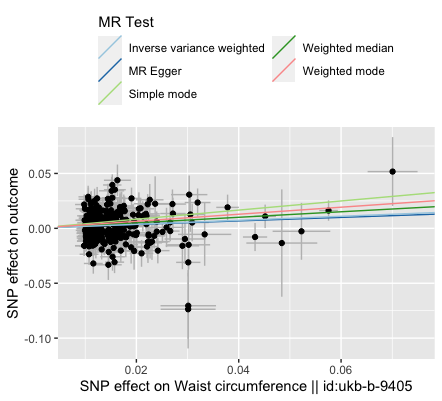


Scatterplot of Mendelian randomization (MR) estimates of genetic risk of WC on Rapid3(D), CKDi25(E) and CKD(F).

G
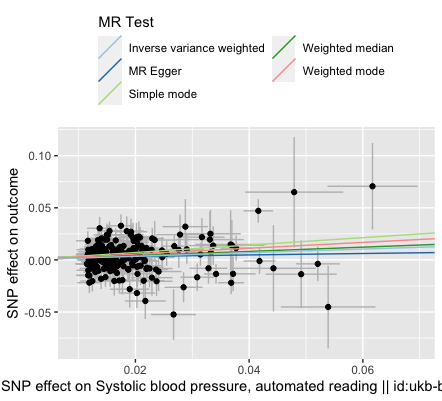


H
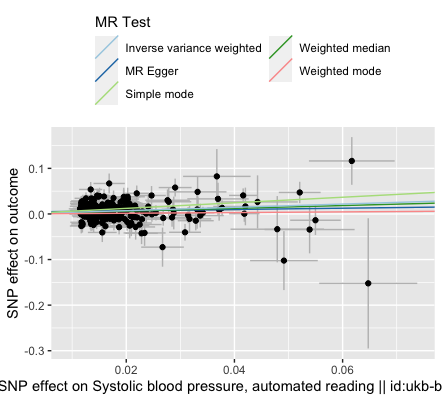


I
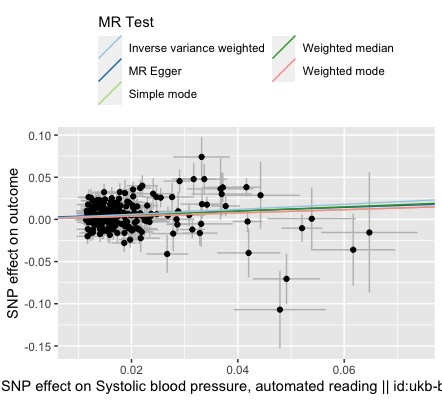


Scatterplot of Mendelian randomization (MR) estimates of genetic risk of SBP on Rapid3(G), CKDi25(H) and CKD(I).

J
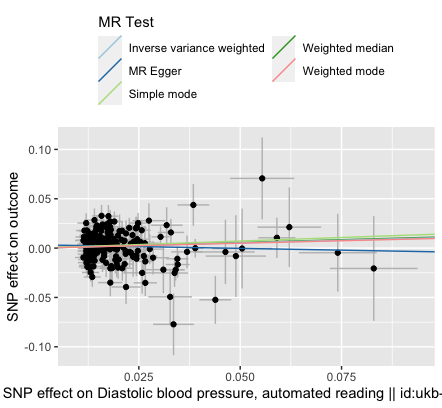


K
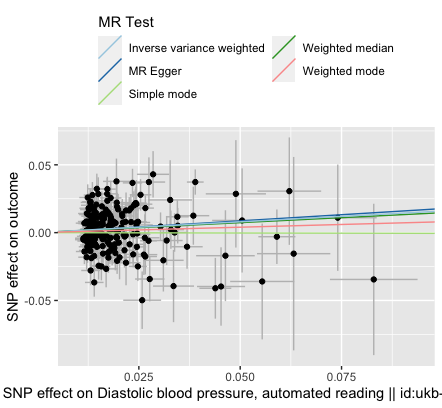


Scatterplot of Mendelian randomization (MR) estimates of genetic risk of SBP on Rapid3(J) and CKD(K).
